# Calibration and analysis of discrete element simulation parameters of Chinese cabbage seeds

**DOI:** 10.1371/journal.pone.0270415

**Published:** 2022-06-24

**Authors:** Shengsheng Wang, Lu Mengqing, Xingyi Liu, Jiangtao Ji, Pan Chen

**Affiliations:** 1 College of Agricultural Equipment Engineering, Henan University of Science and Technology, Luoyang, China; 2 Collaborative Innovation Centre of Machinery Equipment Advanced Manufacturing of Henan Province, Luoyang, China; Jamia Millia Islamia A Central University, INDIA

## Abstract

**Objective:**

To improve the accuracy of parameters used in discrete element simulation test of Chinese cabbage seeds harvesting process.

**Methods:**

Firstly, the key physical parameters of Chinese cabbage seeds were measured. According to the results, the discrete element simulation model was established and the value range of simulation test parameters was determined. Then, the actual repose angle of Chinese cabbage seeds was obtained by physical accumulation test using bottomless conical cylinder lifting method. Plackett-Burman test, steepest climb test, Box-Behnken test and parameter optimization test were carried out in sequence with the actual angle of repose as the response value. Finally, the obtained parameters are verified.

**Results:**

1. The Plackett-Burman test showed that the seed-seed rolling friction coefficient, the seed-steel rolling friction coefficient, the seed-seed static friction coefficient, and the seed-steel static friction coefficient had significant effects on the repose angle of Chinese cabbage seeds (*P<0*.*05*). 2. The optimization test showed that the seed-seed rolling friction coefficient was 0.08, the seed-steel rolling friction coefficient was 0.109, the seed-seed static friction coefficient was 0.496, and the seed-steel static friction coefficient was 0.415. 3. The validation test showed that the repose angle of Chinese cabbage seeds under such parameter was 23.62°, and the error with the repose angle of the physical test was 0.73%.

**Conclusion:**

The study show that the discrete element simulation parameters of Chinese cabbage seeds model and calibration are reliable, which can provide reference for the discrete element simulation of Chinese cabbage seeds.

## 1 Introduction

With the continuous development of seed industry, the vegetable planting area in China is increasing year by year. As a typical representative of cruciferous vegetable seeds, Chinese cabbage seeds has an annual planting area of about 13,000 hectares and an annual output of about 20,000 tons, playing an important role in the development of seed industry [1–3]. Seed harvest is an important part of Chinese cabbage seeds production, and mechanized harvest is an important way to improve the planting efficiency of Chinese cabbage seeds. Due to the traditional test methods cannot accurately analyze the movement process of Chinese cabbage seeds in harvesting machinery, the research on Chinese cabbage seeds mechanized harvesting equipment is relatively backward compared with other crops. This seriously restricted the mechanization process of Chinese cabbage seeds industry [4, 5].

In recent years, EDEM software based on discrete element method has been widely applied to study the movement process of bulk materials, providing a new idea for the study of agricultural material dynamics [6–8]. Hao et al. [9], Liu et al. [10], Yu et al. [11] established discrete element models of irregular spherical granular materials such as oil sunflower seeds, rice and panax notoginseng seeds by combining 3D scanning reverse modeling technology with EDEM software, which improved the accuracy of the simulation model. Lu et al. [12] calibrated the main contact parameters of discrete elements of rice bud seeds with different moisture content. Horabik et al. [13] proposed a method to determine the recovery coefficient of discrete element seed model under different moisture content. Ma et al. [14] designed a device that can simultaneously measure the repose angle and stacking angle of materials, and calibrated the parameters of discrete element model of alfalfa seeds. Through EDEM software simulation of the test of repose angle, relevant scholars have carried out systematic calibration studies on the parameters of discrete element models of wheat [15, 16], corn [17–19], soybean [20], ice grass [21], yam [22], castor [23]. However, the physical parameter calibration and analysis of discrete element simulation model of Chinese cabbage seeds are rarely reported.

In order to solve the difficulty of measuring contact parameters between Chinese cabbage seeds and different materials and the lack of parameters used in discrete element simulation test of harvesting process, this paper took Chinese cabbage seeds as the research object, adopted the method of combining physical experiment and simulation experiment, and applied EDEM software to calibrate the contact parameters of Chinese cabbage seeds. With the repose angle as the response value, the repose angle of Chinese cabbage seeds was measured by image processing method. Plackett-Burman test, steepest climb test and Box-Behnken test were carried out in sequence to calibrate and calibrate the discrete element simulation parameters of Chinese cabbage seeds, and finally to determine the better discrete element simulation parameters of Chinese cabbage seeds. This study can provide reliable discrete element model parameters for the simulation of mechanized harvesting operation and equipment development of Chinese cabbage seeds.

## 2 Materials and methods

In this study, Chinese cabbage seeds “JinCai No. 3”planted in the green breeding base of JiYuan City, Henan Province were used as experimental objects.

### 2.1 Determination of basic physical parameters

**Fig 1:**
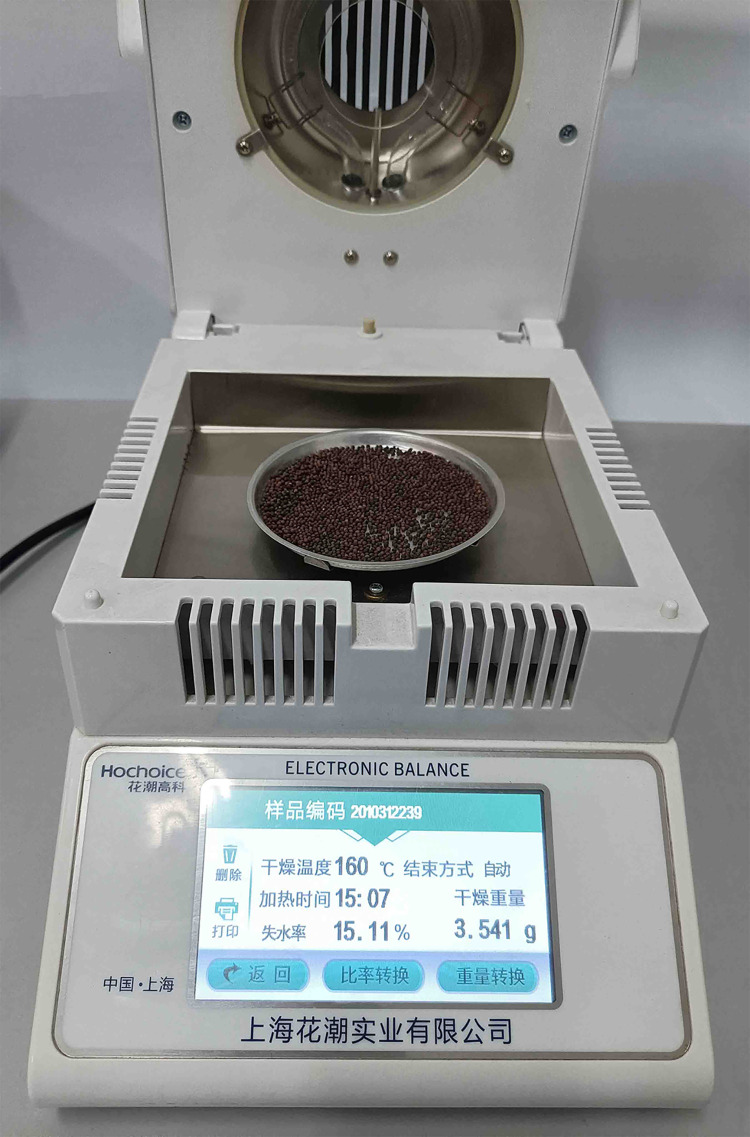
Determination of moisture content of Chinese cabbage seeds.

Firstly, the basic physical characteristics of Chinese cabbage seeds which were easy to be measured were measured. 1000 Chinese cabbage seeds were randomly selected, and the thousand-seed weight was measured by an electronic balance with an accuracy of 0.1*g*. 1000 seeds were divided into 5 groups and weighed and averaged. The external dimensions of seeds were measured with a digital display vernier caliper with a measuring accuracy of 0.02*mm*, and the volume of seeds was measured with a measuring cylinder with a measuring accuracy of 1*mL*. The moisture content is measured by fast moisture tester (Model: SFY-001), as shown in Fig 1.

After 10 repeated tests, the average thousand-seed weight, dimension, density and moisture content of Chinese cabbage seeds were measured as shown in Table 1.

**Table 1:** Basic physical properties of Chinese cabbage seeds.

Parameter	Number
Dimensions (length × width × thickness) /*mm*×*mm*×*mm*	2.10×1.98×1.75
Thousand-seed weight /*g*	3.3
Density /(kg·m^-3^)	803.2
Moisture content /%	15.16

#### 2.1.1 Poisson’s ratio

**Fig 2:**
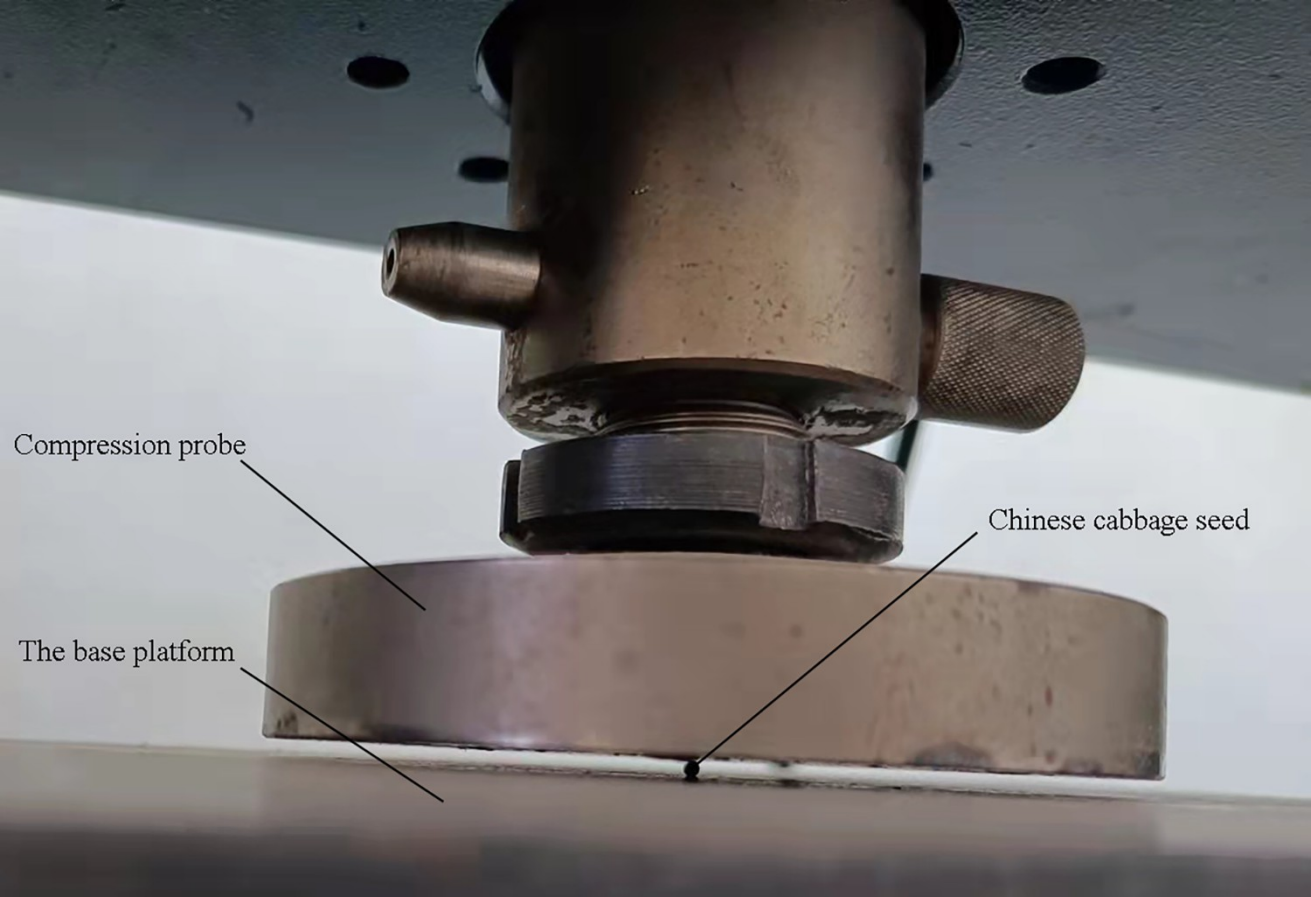
Compression test of Chinese cabbage seed.


30 Chinese cabbage seeds were randomly selected from the above samples, and their original sizes in the directions of length (transverse) and thickness (longitudinal) were recorded. Universal testing machine (Model: HY-0580) was used to apply pressure along the direction of Chinese cabbage seeds thickness at the loading speed of 0.01*mm/s* until the Chinese cabbage seed broke, as shown in Fig 2. The test stopped automatically when Chinese cabbage seeds were crushed and broken and the pressure suddenly decreased. The deformation amount of Chinese cabbage seeds’ longitudinal normal strain was read and the deformation amount of transverse normal strain was measured by using absolute origin digital caliper [24]. Poisson’s ratio of Chinese cabbage seeds was calculated according to Formula (1), and the average result of repeated 30 times was 0.353.

$$\mu =|\frac{\Delta l}{\Delta h}|=|\frac{L_{2}-L_{1}}{H_{2}-H_{1}}|$$ (1)

Where, *μ* is Poisson’s ratio; Δ*l* is the transverse deformation of seed, *mm*; Δ*h* is the longitudinal deformation of seed, *mm*; *L*_1_ is the length of seed before loading, *mm*; *L*_2_ is the length of seed after loading, *mm*; *H*_1_ is the thickness of seed before loading, *mm*; *H*_2_ is the thickness of seed after loading, *mm*.

#### 2.1.2 Modulus of elasticity

Elastic modulus is a measure of a material’s ability to resist elastic deformation. During the test, Chinese cabbage seeds were naturally placed on the platform of the universal testing machine. A load was applied to Chinese cabbage seeds at a loading speed of 5*mm/min* to obtain the load-displacement relationship curve of the seeds. The above tests were repeated for 30 Chinese cabbage seeds respectively. According to Formula (2), the average elastic modulus is 136.465MPa.


$$E=\frac{\sigma }{\varepsilon }$$ (2)

Where, *E* is the elastic modulus of Chinese cabbage seeds, Pa; *σ* is the maximum compressive stress, Pa;**Error! Objects cannot be created from editing field codes.** is linear strain.

### 2.2 Contact parameter measurement

#### 2.2.1 Static friction coefficient measurement

**Fig 3:**
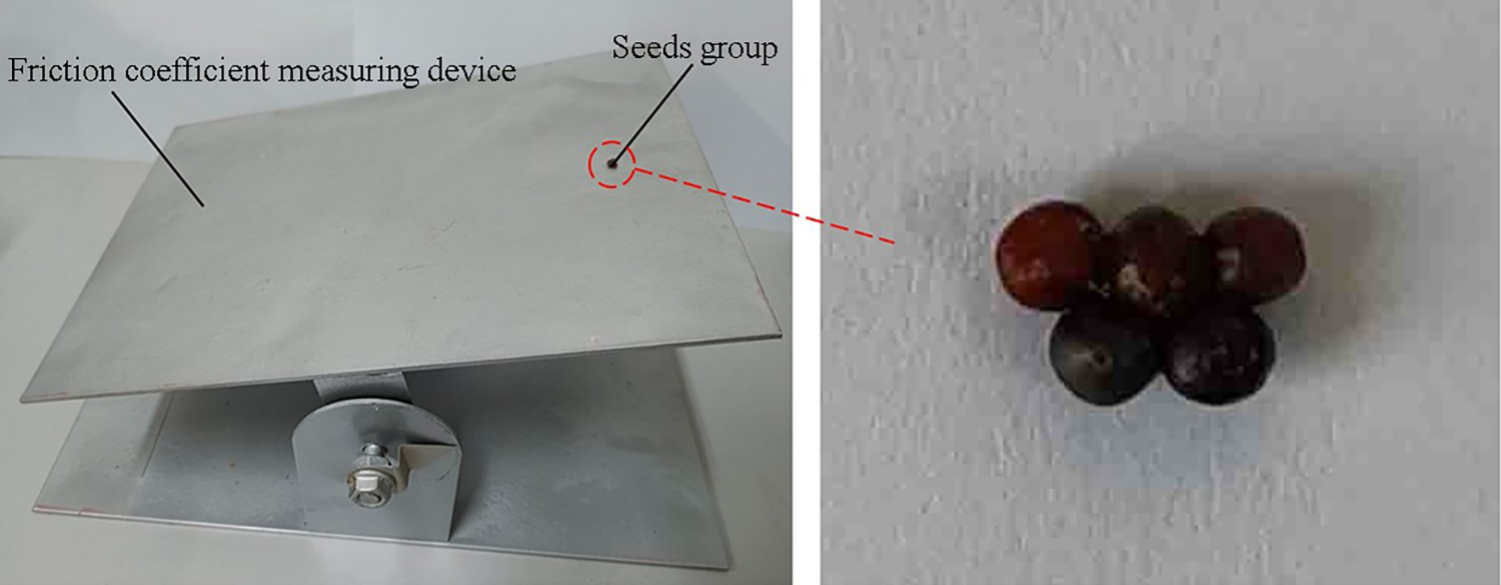
Determination of friction coefficient of Chinese cabbage seeds.

In this study, the inclined plane method [25] was used to measure the static friction coefficient of Chinese cabbage seeds according to the self-made friction coefficient measuring device, as shown in Fig 3. During the test, the device was slowly rotated so that the inclination angle of the inclined plane gradually increased. When the seed slides downward on the inclined plane, the inclination angle of the inclined plane is the sliding friction angle, denoted as *φ*. Chinese cabbage seeds are bulk materials with small size and easy to tumble. In order to prevent Chinese cabbage seeds from tumbling, 5 Chinese cabbage seeds were glued together to form a seeds group and repeated the test to obtain a more accurate static friction coefficient. The static friction coefficient of Chinese cabbage seeds is calculated according to Formula (3).


f=tanφ (3)

It is difficult to measure the coefficient of static friction between Chinese cabbage seeds directly because the surface of Chinese cabbage seeds is irregular. In order to facilitate measurement, according to literature [26, 27], the test seeds were made into seed boards with adhesive and pasted on the test plane, so that the cabbage seeds could be arranged as closely as possible. Place the Chinese cabbage seeds’ group on the seed board, slowly turn the device until the Chinese cabbage seeds’ group appears to slide on the seed board, stop rotating and record the inclined plane angle at this time. The experiments were repeated for 30 times in each group, and the average value was obtained. The average value of static friction coefficient between Chinese cabbage seeds and steel was 0.392, and the average value of static friction coefficient between Chinese cabbage seeds was 0.441.

#### 2.2.2 Rolling friction coefficient measurement

The testing method of rolling friction coefficient is similar to that of static friction coefficient. The rolling friction coefficient between tested objects is calculated by using the angle of single seed rolling in the process of lifting the inclined plane of the measuring device. The average coefficient of rolling friction between Chinese cabbage seeds and steel was 0.083 and 0.096, respectively, after 30 repeated tests.

### 2.3 Determination of seed actual repose angle

**Fig 4:**
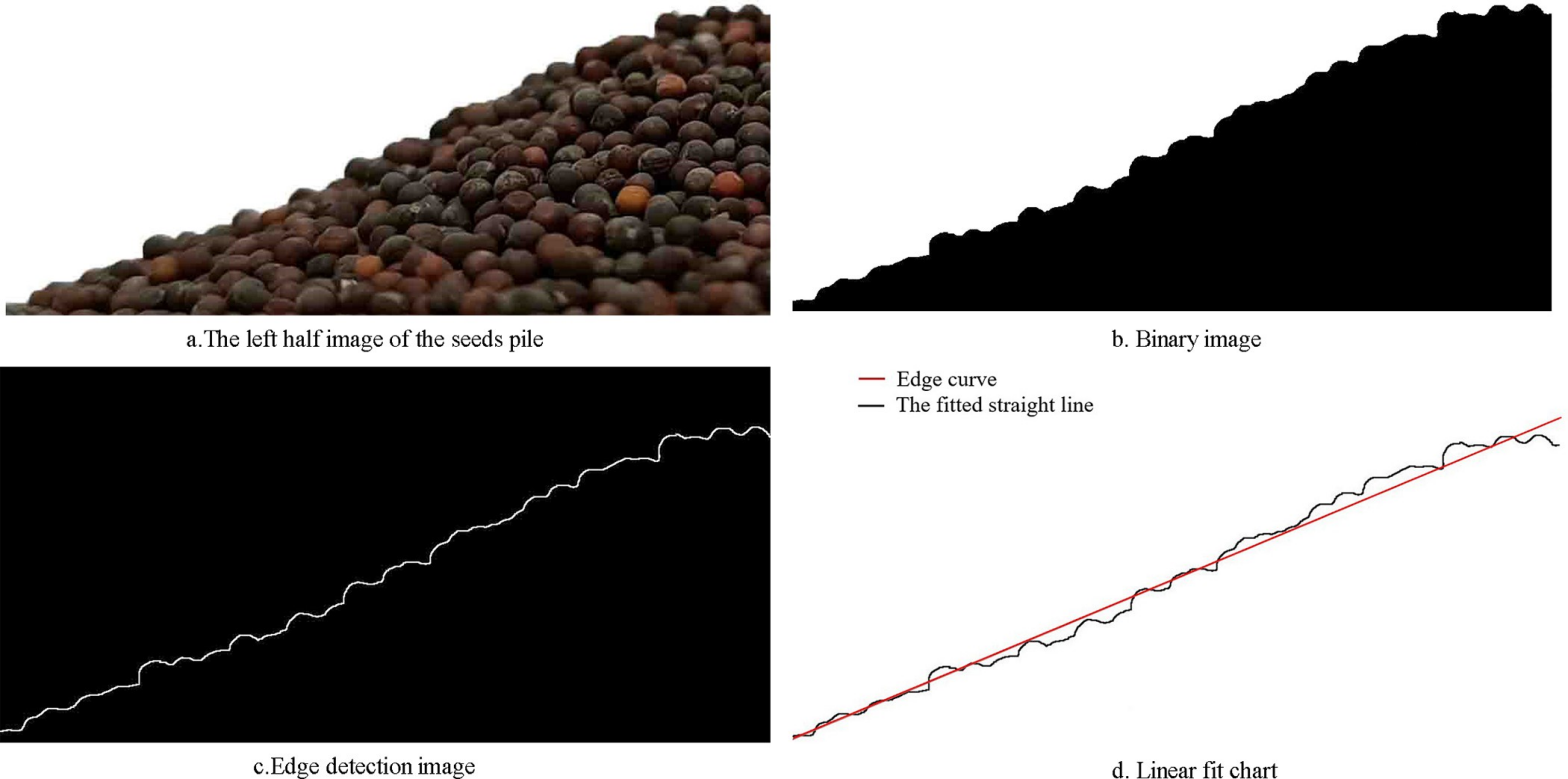
Image processing of Chinese cabbage seeds’ pile.

Chinese cabbage seeds’ pile was obtained by bottomless conical cylinder lifting method, and the actual repose angle was measured [28]. Before the test, remove impurities, broken seeds and damaged seeds from the material. The measured moisture content of dried seeds was 15.16%. During the test, the bottom plate is placed on the horizontal test table, and the conical cylinder is placed on the bottom plate with the small mouth facing down. The conical cylinder was filled with Chinese cabbage seeds and was slowly raised perpendicular to the bottom plate to form a stable seeds pile. The frontal image of the seed pile was taken with a high-definition camera. The left half image of the seeds pile as shown in Fig 4A. The seeds pile image was processed with Matlab for grayscale processing and binarization processing (Fig 4B), then the extraction and fitting of image edge pixels are obtained (Fig 4C). Finally, the unilateral repose angle of Chinese cabbage seeds is obtained.

## 3 Simulation model and parameter setting

### 3.1 Construction of simulation model

**Fig 5:**
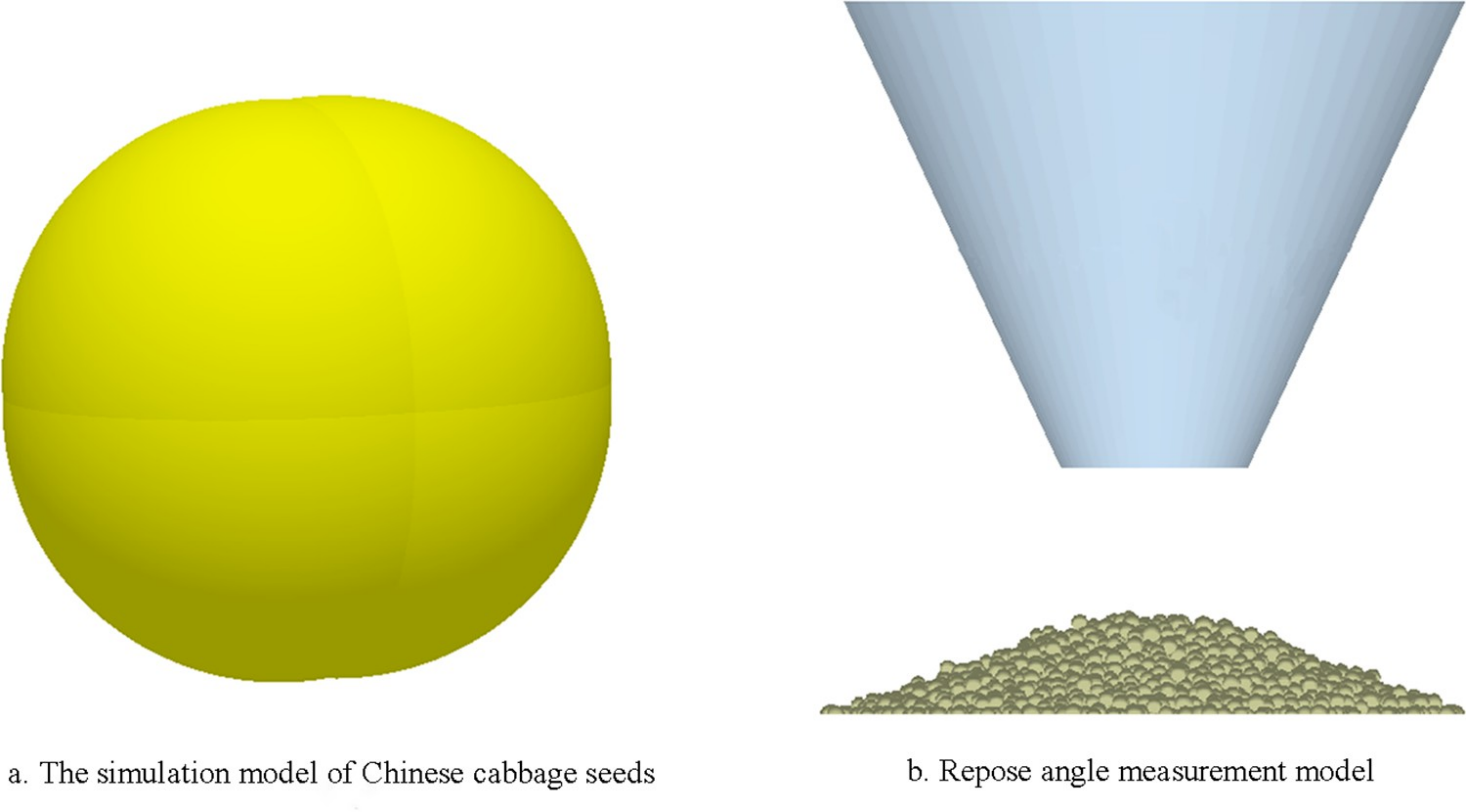
Measurement model of simulation test of repose angle.

The discrete element simulation software is EDEM 2018, and Hertz-Mindlin (No slip) contact model is used for particle to particle and particle to geometry contact model in simulation calculation. The Chinese cabbage seeds’ model was established in discrete element software, and parameters were set according to the triaxial sizes measured in the test. The Chinese cabbage seeds model finally generated was composed of four spheres with a radius of 0.9mm, as shown in Fig 5A. The 3D model of conical cylinder is built in SolidWorks according to the actual size and saved in STL format, and then imported into EDEM software. The steel plate model was directly generated by EDEM software, and the material property was set as steel. The Polygon virtual particle plane was established in the diameter above the conical cylinder to generate Chinese cabbage seeds’ particles. The particles generation mode adopts dynamic generation mode, the particles generation rate was 0.1kg/s, and 0.1kg particles were generated in total. In order to give consideration to the simulation efficiency and the reliability of the simulation results, the size of the generated particles was set as 50% of the initial radius particles, and 25% of the initial radius particles were 0.9 and 1.1 times respectively. The total simulation time was 5s, the time step was 0.01s, and the mesh size was 3 times of the minimum particle radius [29].

At the beginning of the simulation, seeds were generated from the virtual particle plane at the diameter above the conical cylinder, and began to fall free with the influence of gravity. After 1s, all seeds were generated. After the seeds reached a stable state, the conical cylinder moves vertically upward at a speed of 0.02m/s, and the seeds fall down on the steel plate under the action of gravity. Until all seeds stop moving and a stable seeds pile is formed, as shown in Fig 5B, the vertical view of the seed pile in different directions is captured and the repose angle is measured.

### 3.2 Simulation parameter setting

The main contact parameters of Chinese cabbage seeds and steel were used as the test factors, and two virtual variables were introduced to estimate the error. According to relevant research literature and simulation pre-test in this study [30, 31], the levels of these test factors were determined, as shown in Table 2.

**Table 2:** Simulation parameters setting of Chinese cabbage seeds.

Parameters	Low-level	High-level
Poisson’s ratio of Chinese cabbage seed *A*	0.2	0.5
Elastic modulus of Chinese cabbage seed *B*/MPa	50	220
Chinese cabbage seed density *C*/(kg·m^-3^)	600	1000
Seed-Seed collision restitution coefficient *D*	0.35	0.75
Seed-Seed static friction coefficient *E*	0.3	0.6
Seed-Seed rolling friction coefficient *F*	0.036	0.136
Seed- Steel collision restitution coefficient *G*	0.3	0.7
Seed-Steel static friction coefficient *H*	0.28	0.52
Seed-Steel rolling friction coefficient *J*	0.034	0.126
Virtual variable *K*	-1	1
Virtual variable *L*	-1	1

## 4 Simulation test results and analysis

### 4.1 Plackett-Burman screening test and significance analysis

In this study, Plackett-Burman module of Design-Expert software was used to screen out the parameters that have significant influence on the index by taking the repose angle of Chinese cabbage seeds as the test index [15]. Three centers were set for the screening test, with a total of 15 groups of tests. Each group of tests was repeated three times to calculate the average. The test results were shown in Table 3.

**Table 3:** Design and results of Plackett-Burman test scheme.

No.	*A*	*B*	*C*	*D*	*E*	*F*	*G*	*H*	*J*	*K*	*L*	Repose angle /(°)
1	1	1	-1	1	1	1	-1	-1	-1	1	-1	25.96
2	-1	1	1	-1	1	1	1	-1	-1	-1	1	24.92
3	1	-1	1	1	-1	1	1	1	-1	-1	-1	24.79
4	-1	1	-1	1	1	-1	1	1	1	-1	-1	23.15
5	-1	-1	1	-1	1	1	-1	1	1	1	-1	33.71
6	-1	-1	-1	1	-1	1	1	-1	1	1	1	25.15
7	1	-1	-1	-1	1	-1	1	1	-1	1	1	17.48
8	1	1	-1	-1	-1	1	-1	1	1	-1	1	30.25
9	1	1	1	-1	-1	-1	1	-1	1	1	-1	18.54
10	-1	1	1	1	-1	-1	-1	1	-1	1	1	14.87
11	1	-1	1	1	1	-1	-1	-1	1	-1	1	19.71
12	-1	-1	-1	-1	-1	-1	-1	-1	-1	-1	-1	14.71
13	0	0	0	0	0	0	0	0	0	0	0	24.64
14	0	0	0	0	0	0	0	0	0	0	0	22.86
15	0	0	0	0	0	0	0	0	0	0	0	24.14

The Design-Expert 10 software was used to conduct variance analysis on the test results, and the significance results of each simulation parameter were obtained, as shown in Table 4. As can be seen from Table 4, The influence of seed-seed rolling friction coefficient and seed-steel rolling friction coefficient on the angle of repose in simulation experiment is extremely significant *(P<0*.*01)*. The seed-seed static friction coefficient and seed-steel static friction coefficient had significant effects on the angle of repose *(P<0*.*05)*. Other simulation test parameters have little effect on the angle of repose *(P>0*.*05)*.

**Table 4:** Analysis of significance of parameters in Plackett-Burman test.

Element	Standardization effects	Sum of squares	Contribution rate/%	*F* values	*P* values
*A*	0.037	0.004	0.001	0.003	0.9625
*B*	0.36	0.38	0.098	0.25	0.6536
*C*	-0.027	0.002	0.006	0.001	0.9727
*D*	-1.00	2.98	0.77	1.93	0.2593
*E*	2.77	23.02	5.94	14.87	0.0308*
*F*	9.39	264.33	68.20	170.80	0.0010 * *
*G*	-0.86	2.24	0.58	1.44	0.3156
*H*	2.54	19.41	5.01	12.54	0.0383*
*J*	4.63	64.31	16.59	41.56	0.0076 **

Note

** represents extremely significant influence *(P<0*.*01)*

* represents significant influence *(0*.*01<P<0*.*05)*.

### 4.2 Steepest climb test

Based on the analysis of the Plackett-Burman test results, the steepest climb test was performed on the four selected factors (seed-seed rolling friction coefficient, seed-steel rolling friction coefficient, seed-seed static friction coefficient, seed-steel static friction coefficient) that contributed significantly. The relative error between actual angle of repose and simulation angle of repose is calculated to determine the optimal range of simulation parameters. In the simulation process, the intermediate level values were taken for the parameters that contributed less to the repose angle: Poisson’s ratio of Chinese cabbage seeds 0.35, elastic modulus of Chinese cabbage seeds 135MPa, density of Chinese cabbage seeds 800kg·m^-3^, seed-seed collision restitution coefficient 0.55and seed- steel collision restitution coefficient 0.5. The design and results of the steepest climb test are shown in Table 5.

**Table 5:** Design and results of climbing test.

No.	*F*	*J*	*E*	*H*	Repose angle *θ*/(°)	Relative error /%
1	0.036	0.034	0.3	0.28	15.33	34.63
2	0.061	0.057	0.375	0.34	19.78	15.65
3	0.086	0.08	0.45	0.4	22.5	4.05
4	0.111	0.103	0.525	0.46	28.36	20.94
5	0.136	0.126	0.6	0.52	30.01	27.97

The results showed that the relative error of repose angle was the smallest in the third group of test, and the optimal interval was determined to be near the third group of parameters. Therefore, the third group of parameters was taken as the intermediate level, and the second and fourth group of parameters were taken as the low level and high level respectively for the subsequent Box-Behnken test.

### 4.3 Box-Behnken test

In Design-Expert software, group 3 in the steepest climbing test was taken as the center point (0), group 2 and group 4 as the low level (-1) and high level (+1) respectively. The Design scheme and results of Box-Behnken test are shown in Table 6. In the simulation test, other non-significant parameters were set according to the parameters used in the steepest climb test.

**Table 6:** Test scheme and results.

No.	*F*	*J*	*E*	*H*	Repose angle /(°)
1	-1	-1	0	0	23.03
2	1	-1	0	0	27.89
3	-1	1	0	0	23.6
4	1	1	0	0	29.44
5	0	0	-1	-1	22.06
6	0	0	1	-1	26.34
7	0	0	-1	1	24.68
8	0	0	1	1	25.67
9	-1	0	0	-1	23.57
10	1	0	0	-1	28.8
11	-1	0	0	1	23.65
12	1	0	0	1	29.29
13	0	-1	-1	0	22.39
14	0	1	-1	0	27.07
15	0	-1	1	0	25.52
16	0	1	1	0	26.3
17	-1	0	-1	0	21.92
18	1	0	-1	0	25.68
19	-1	0	1	0	21.04
20	1	0	1	0	28.14
21	0	-1	0	-1	24.23
22	0	1	0	-1	28.65
23	0	-1	0	1	25.38
24	0	1	0	1	28.25
25	0	0	0	0	25.19
26	0	0	0	0	25.34
27	0	0	0	0	24.98
28	0	0	0	0	23.86
29	0	0	0	0	25.03

Through multiple regression fitting of the test results, the second-order regression equation of the angle of repose of the simulation test was obtained:

$$\begin{array}{l} \theta =-2.22-172.04F+24.31J+143.95E-51.02H+312.50FJ+298.21FE+45.76FH\\ -348.21JE-172.99JH-102.81EH+372.02F^{2}+1280.82J^{2}-86.33E^{2}+133.85H^{2} \end{array}$$ (4)

The results of Box-Behnken test variance analysis are shown in Table 7. It can be seen from the analysis results in Table 7 that The coefficients *F*, *J*, *E*, and *J*^*2*^ had extremely significant effects on the repose angle*(P<0*.*01)*, and the coefficients *FE*, *JE*, *EH*, *E*^*2*^ and *H*^*2*^ had significant effects on the repose angle*(0*.*01<P<0*.*05)*, and other coefficients were not significant*(P>0*.*05)*. The angle of repose fitting regression model was *P<*0.0001, and the lack of fit was *P* = 0.2729>0.05, indicating that the model fit well and no lack of fit phenomenon occurred. The determination coefficient *R*^*2*^ = 0.9465, and the correction determination coefficient *Adj-R*^*2*^ = 0.8931, which were all close to 1, and coefficient of variation C.V. = 2.99%. In summary, this regression model is extremely significant and can reliably and truly reflect the real situation, which can be used for further target angle of repose prediction and analysis.

**Table 7:** Variation analysis of Box-Behnken test quadratic model.

Source	Sum of square	Mean square	*F*	*P*
Model	143.18	10.23	17.70	<0.0001* *
*F*	87.64	87.64	151.69	<0.0001* *
*J*	18.43	18.43	31.89	<0.0001* *
*E*	7.07	7.07	12.23	0.0036* *
*H*	0.89	0.89	1.54	0.2347
*FJ*	0.24	0.24	0.42	0.5296
*FE*	2.79	2.79	4.83	0.0454*
*FH*	0.042	0.042	0.073	0.7913
*JE*	3.80	3.80	6.58	0.0224*
*JH*	0.60	0.60	1.04	0.3252
*EH*	2.71	2.71	4.68	0.0482*
*F* ^*2*^	0.55	0.55	0.96	0.3450
*J* ^*2*^	6.54	6.54	11.32	0.0046* *
*E* ^*2*^	4.83	4.83	8.37	0.0118*
*H* ^*2*^	4.76	4.76	8.24	0.0123*
*Residual*	8.09	0.58		
*Lack of Fit*	6.71	0.67	1.94	0.2729
*Pure Error*	1.38	0.35		
*Cor Total*	151.27			

Note

** represents extremely significant influence *(P<0*.*01)*

* represents significant influence *(0*.*01<P<0*.*05)*.

### 4.4 Interaction analysis of regression model

**Fig 6:**
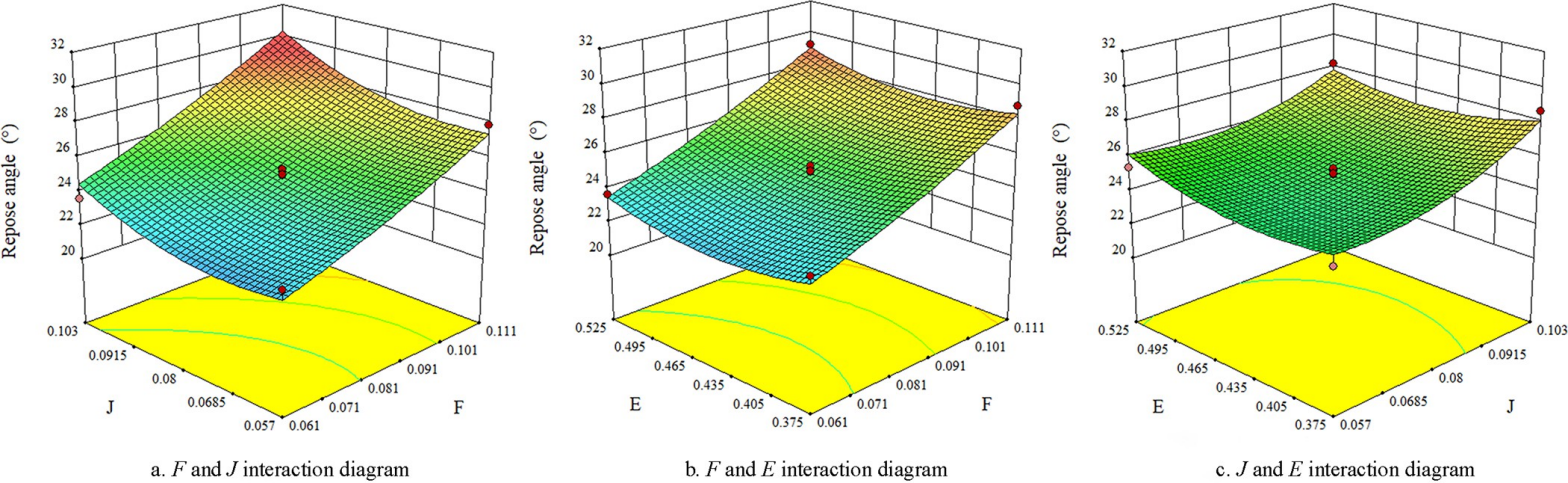
Effect of interaction on repose angle.

With angle of repose as the response value, multiple regression fitting of significant parameters was carried out by using Design-Expert software to further analyze the interaction of various influencing factors on the response value, and the response surface was generated as shown in Fig 5. It can be seen from Fig 6A and 6B that the response surface curve of seed-seed rolling friction coefficient *F* is steeper than that of seed-steel rolling friction coefficient *J* and seed-seed static friction coefficient *E*, indicating that the seed-seed rolling friction coefficient *F* has a more significant impact on the angle of repose. As can be seen from Fig 6C, the response surface curves of seed-steel rolling friction coefficient *J* and seed-seed static friction coefficient *E* have similar trends, indicating that the two factors have basically the same influence on the repose angle.

### 4.5 Parameter optimization and simulation verification

In the optimization module of the Design-Expert 10 software, in order to make the repose angle of simulation test closest to the repose angle of physical test (23.45°), the second-order regression equation (Eq (4)) should be optimized and solved according to the target value of the optimal repose angle (23.45°). A group of optimal parameter combinations is selected which is close to the average value of the measured data in physical experiments. The optimal combination of simulation parameters could be calibrated and calibrated: the seed-seed rolling friction coefficient was 0.08, the seed-steel rolling friction coefficient was 0.109, the seed-seed static friction coefficient was 0.496 and the seed-steel static friction coefficient was 0.415.

**Fig 7:**
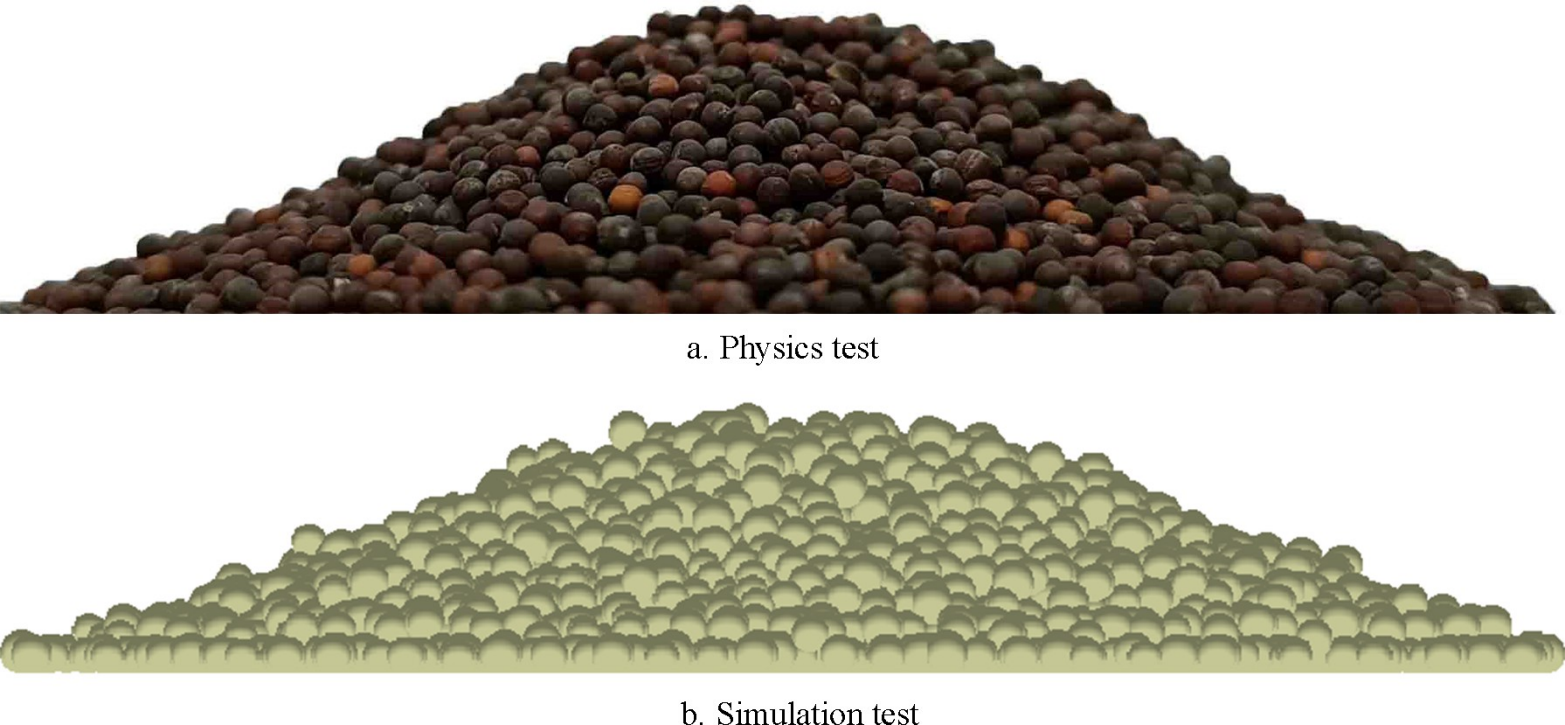
Comparison of physical test and simulation test.

In order to verify the reliability and authenticity of discrete element simulation parameters after calibration of Chinese cabbage seeds, the above parameters were used as EDEM simulation parameters to conduct three simulation tests, and the repose angles of Chinese cabbage seeds were 23.35°, 23.03° and 24.29°, respectively. The relative error between the mean angle of repose of 23.45° in physical test and 23.62° in simulation test is only 0.73%, which further verifies the reliability and authenticity of simulation test. The test comparison is shown in Fig 7.

## 5 Conclusion

1. The basic physical parameters of Chinese cabbage seeds (the shape size, thousand-grain weight, density, moisture content, Poisson’s ratio, elastic modulus) were determined by physical experiments. The self-made friction coefficient measuring device was used to measure: the average values of static friction coefficient and rolling friction coefficient between Chinese cabbage seeds were 0.441 and 0.083 respectively; The average coefficient of static friction and rolling friction between Chinese cabbage seeds and steel were 0.392 and 0.096, respectively.
2. Plackett-Burman test was carried out with physical property parameters measured in physical tests as the basis for parameter selection of simulation tests, and the significant parameters affecting repose angle were screened as follows: seed-seed rolling friction coefficient, seed-steel rolling friction coefficient, seed-seed static friction coefficient, seed-steel static friction coefficient, and further determine the optimal range of significance parameters through the steepest climb test. Box-Behnken test was used to establish the second-order regression equations between the repose angle and the four significant parameters.
3. With the target value of the optimal repose angle (23.45°), the optimal combination of simulation parameters was obtained: the seed-seed rolling friction coefficient, the seed-steel rolling friction coefficient, the seed-seed static friction coefficient and the seed-steel plate static friction coefficient were 0.08, 0.109, 0.496 and 0.415, respectively. The relative error between the repose angle of the optimal parameter combination and the actual physical repose angle is 0.73%, which verifies the reliability of the simulation model parameters.

## Supporting information

S1 Fig EDEM simulation test process. (JPG)

S2 Fig Plackett-Burman test data processing. (JPG)

S3 Fig Plackett-Burman test analysis of variance. (JPG)

S4 Fig Box-Behnken test data processing. (JPG)

S1 File (DOCX)
