# Supplementary figures and images for: Calibration and analysis of discrete element simulation parameters of Chinese cabbage seeds
Source: PLoS One. 2022 Jun 24;17(6):e0270415. doi: 10.1371/journal.pone.0270415 (PMC9232167; doi:10.1371/journal.pone.0270415)

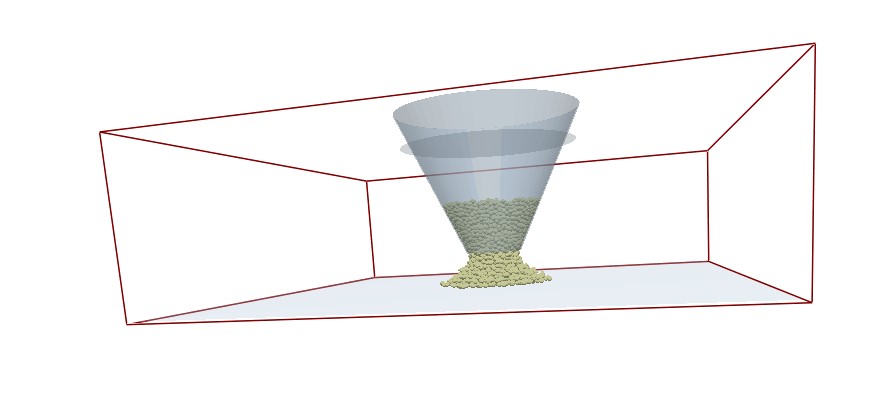

Supplement: S1 Fig — (JPG) [file pone.0270415.s001.jpg]

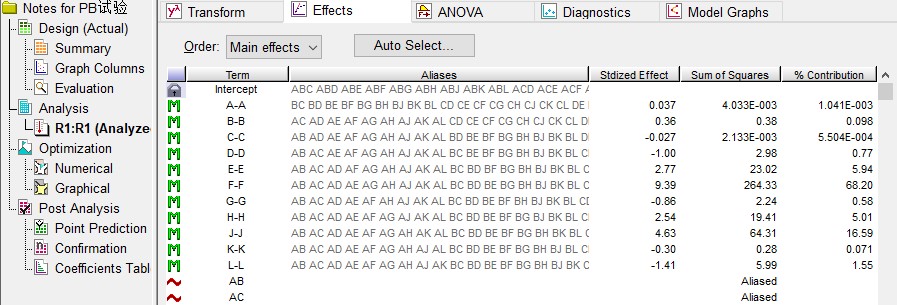

Supplement: S2 Fig — (JPG) [file pone.0270415.s002.jpg]

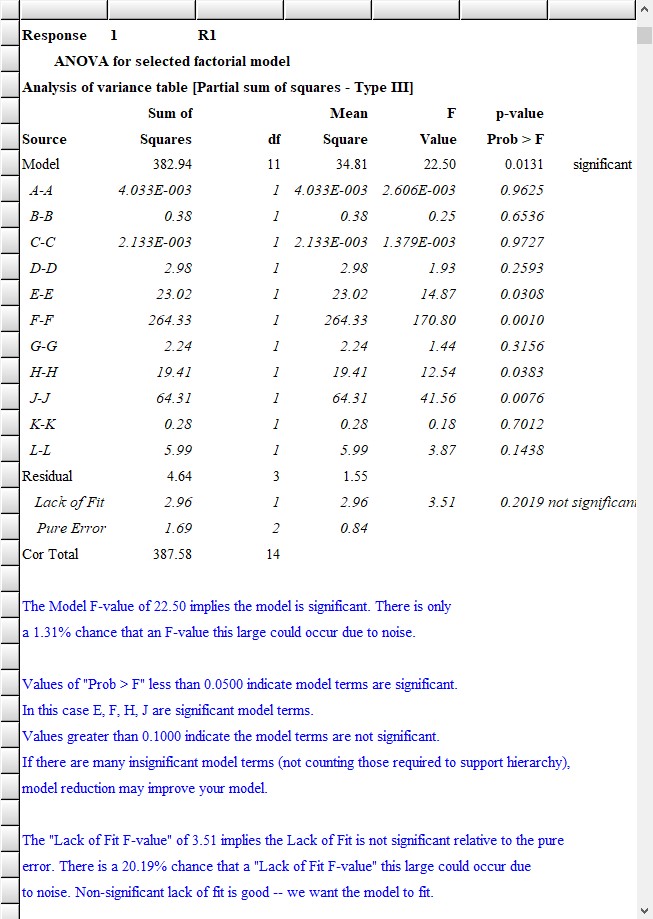

Supplement: S3 Fig — (JPG) [file pone.0270415.s003.jpg]

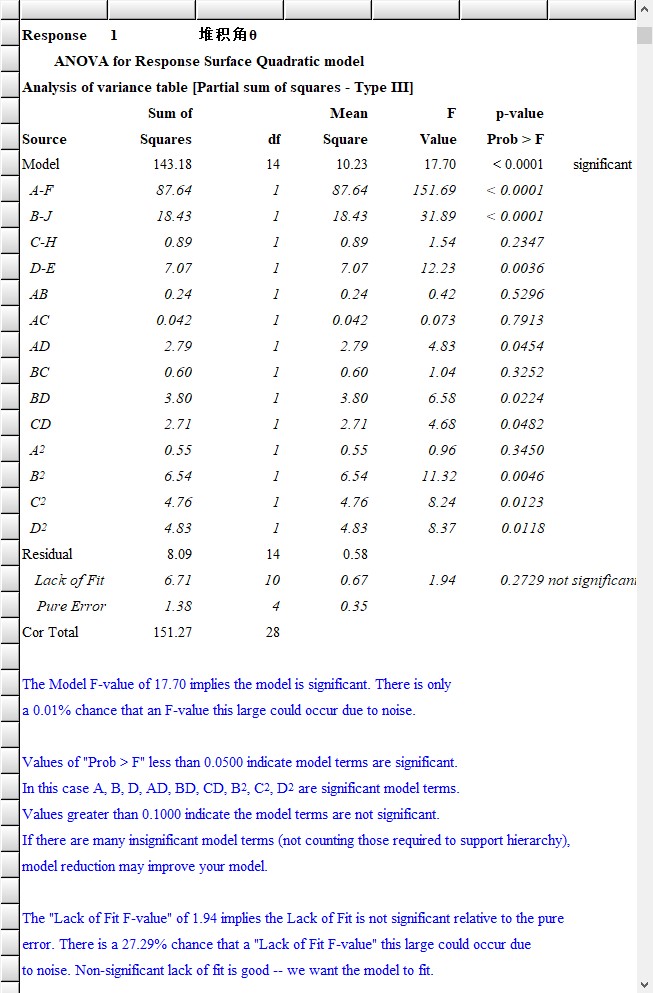

Supplement: S4 Fig — (JPG) [file pone.0270415.s004.jpg]
