# Supplementary material for: Calibration and analysis of discrete element simulation parameters of Chinese cabbage seeds
Source: PLoS One. 2022 Jun 24;17(6):e0270415. doi: 10.1371/journal.pone.0270415 (PMC9232167; doi:10.1371/journal.pone.0270415)

**Supporting Information**

1. EDEM simulation test process：
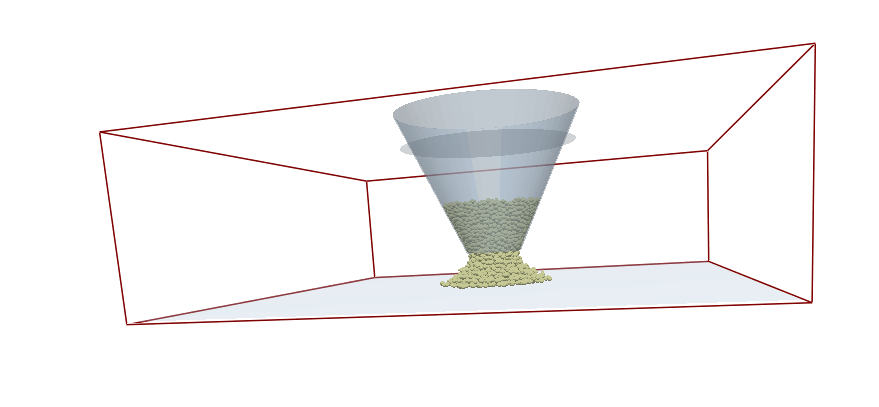


2. Plackett-Burman test data processing：


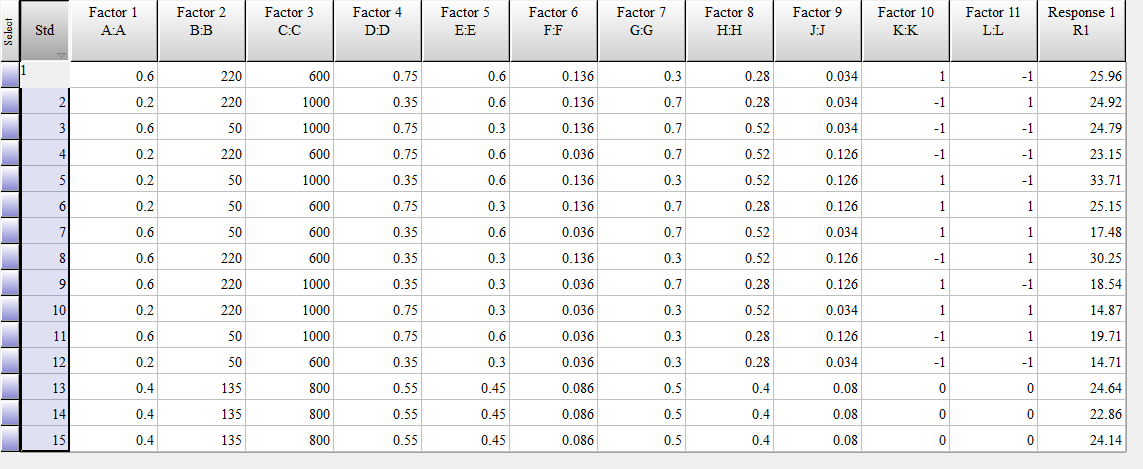


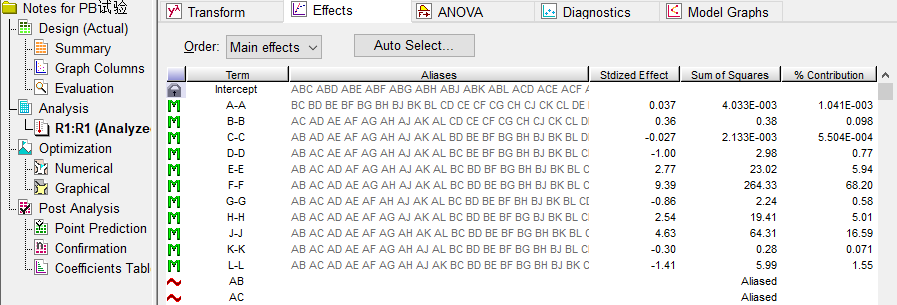


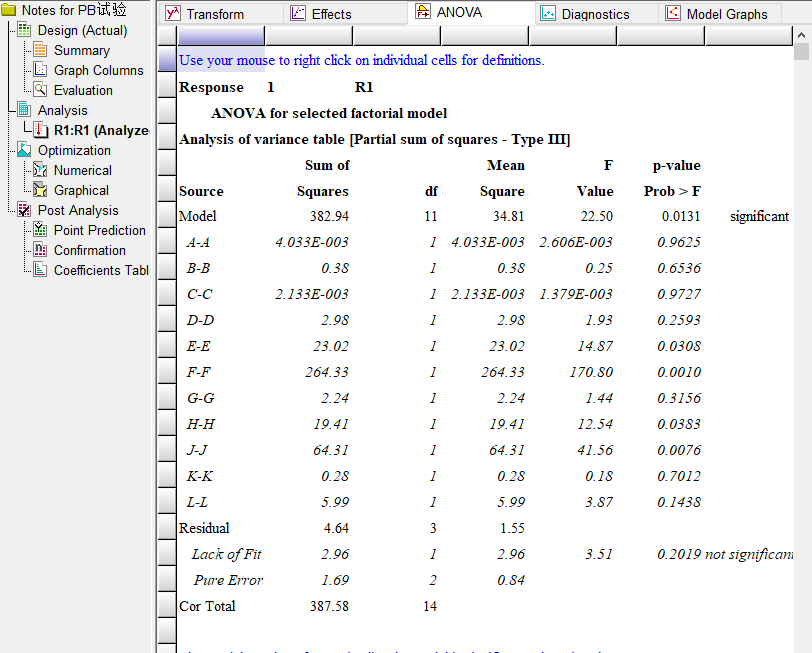


3.Box-Behnken test data processing：


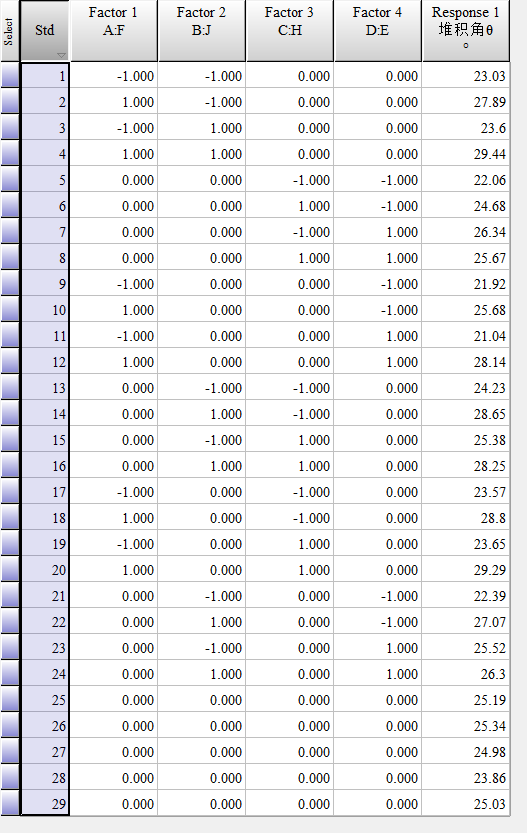

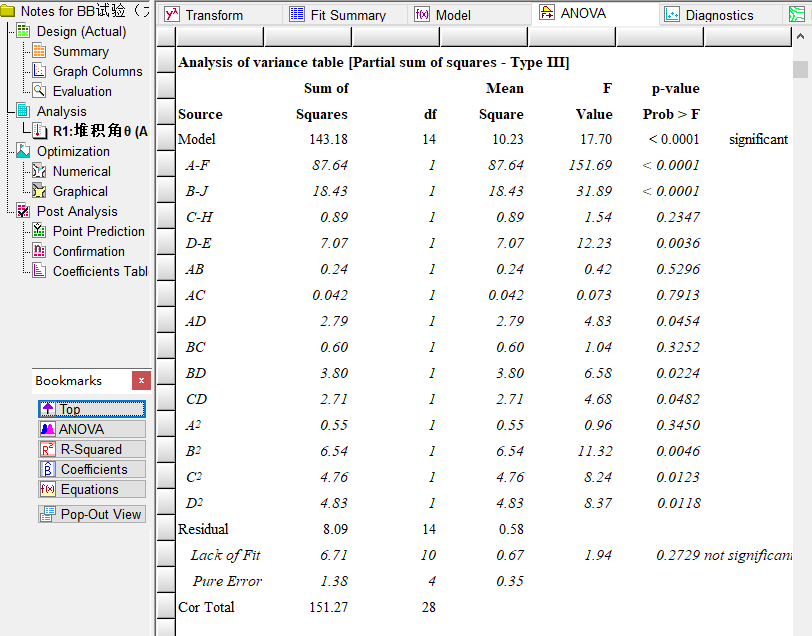


4. Original image：

**
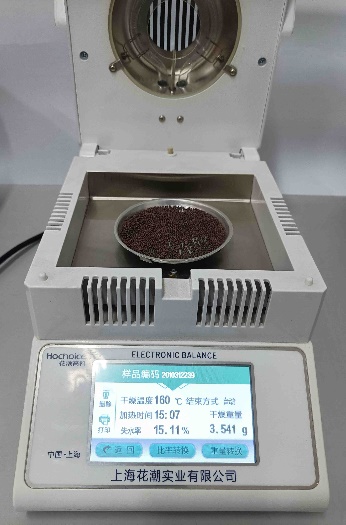

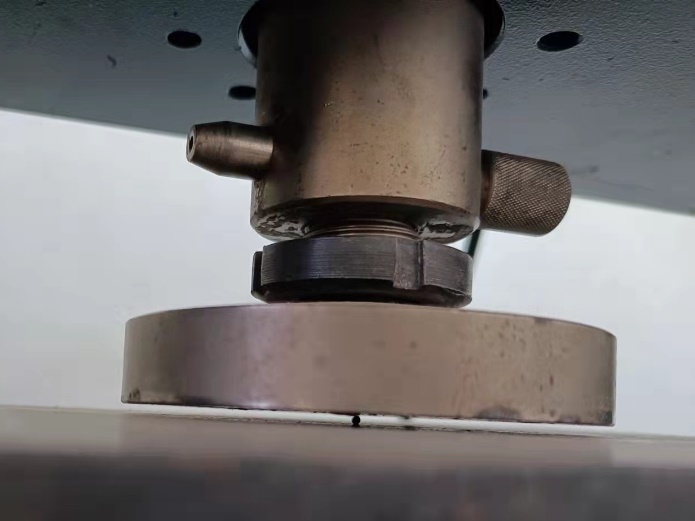
**

**
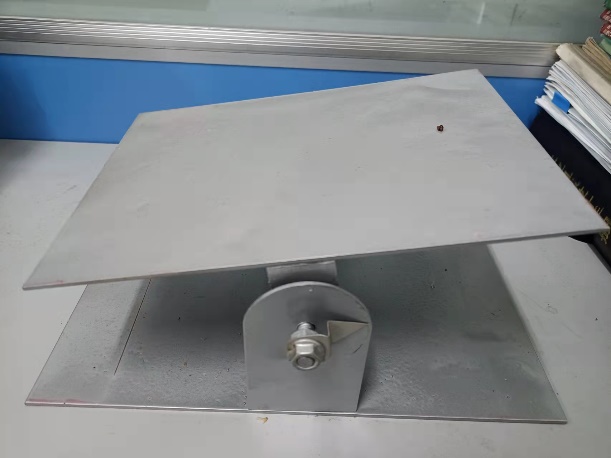
**


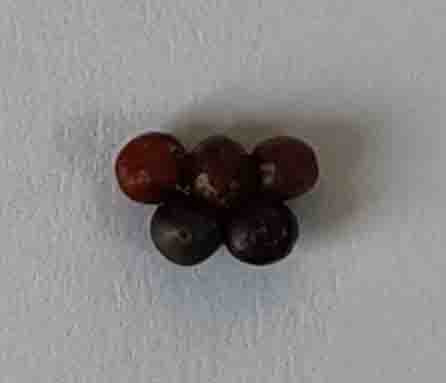

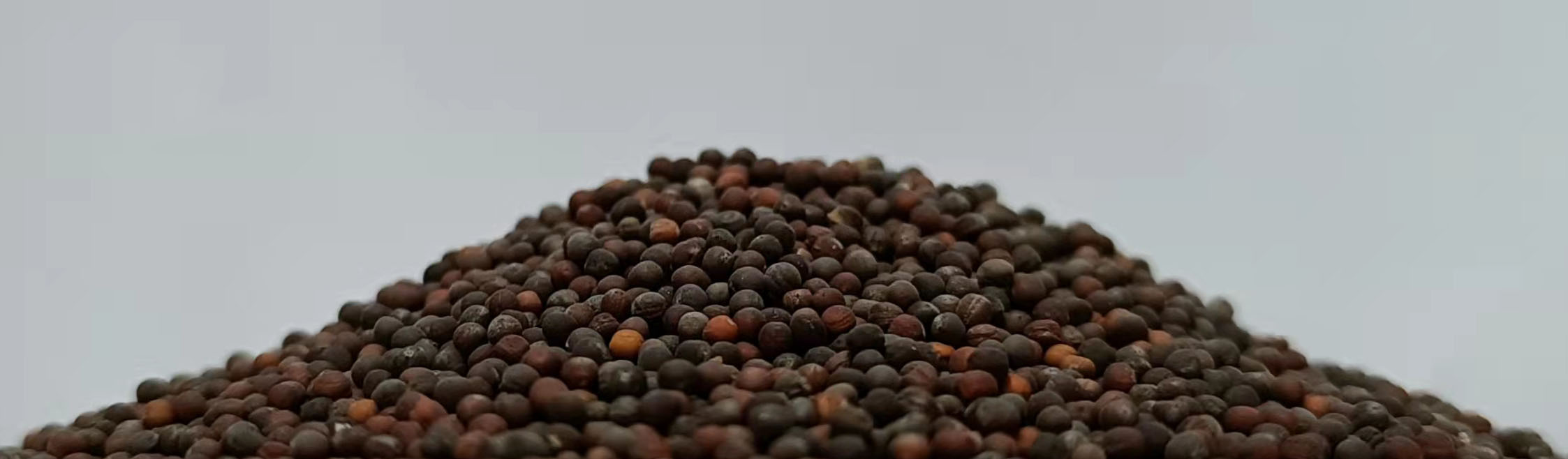


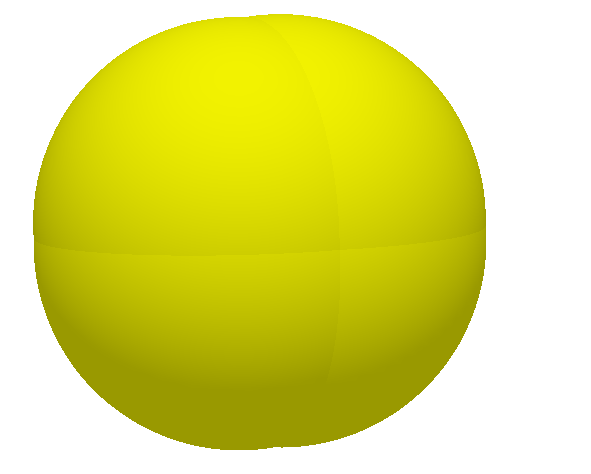


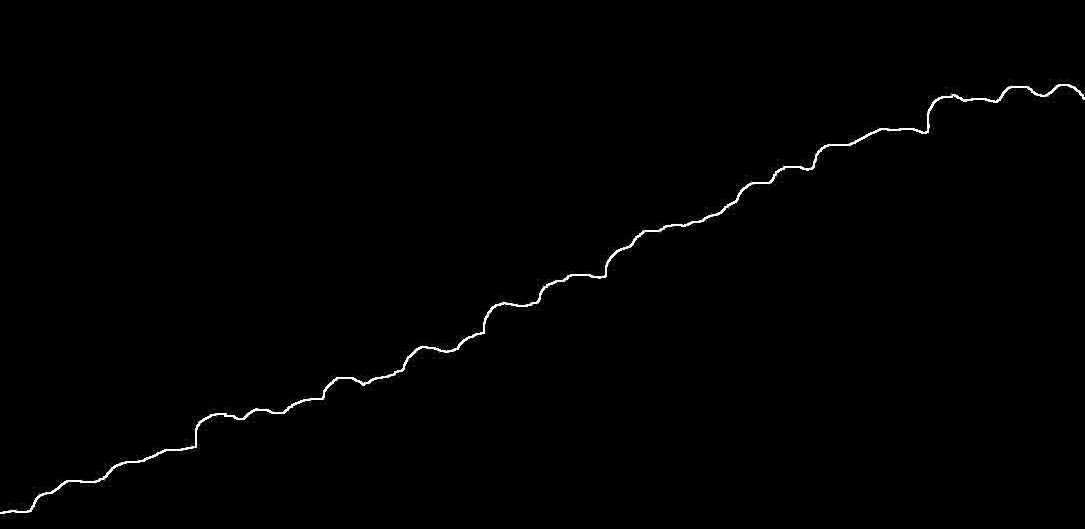


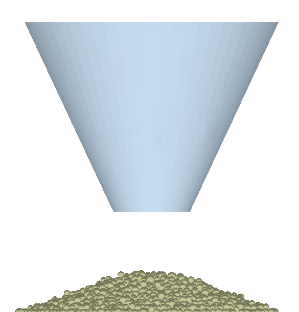


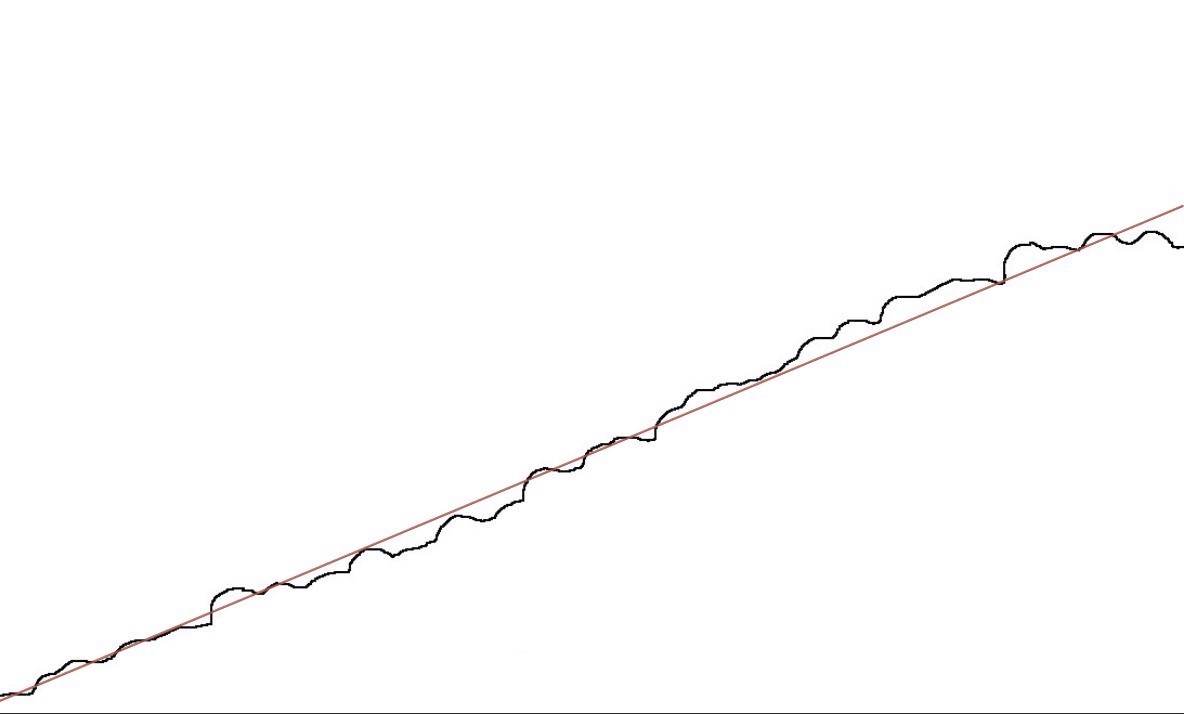


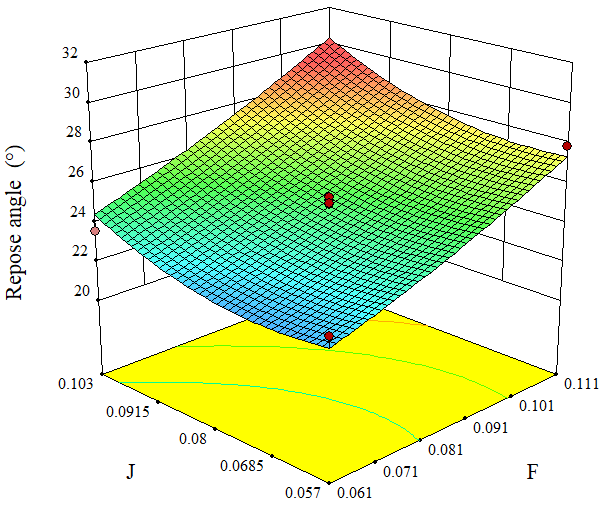

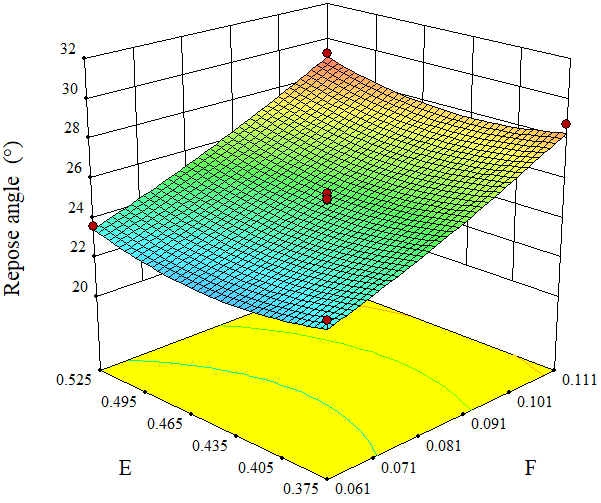


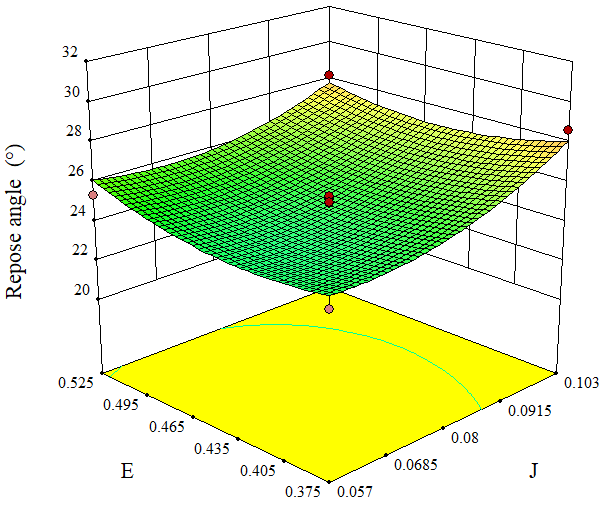

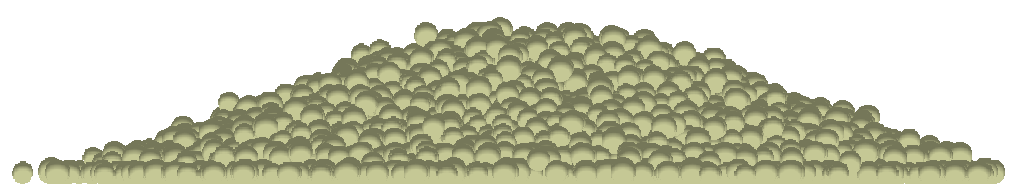

Supplement: S1 File — (DOCX) [file pone.0270415.s005.docx]
